# Patient’s behaviors and missed opportunities for vaccination against seasonal epidemic influenza and evaluation of their impact on patient’s influenza vaccine uptake

**DOI:** 10.1371/journal.pone.0193029

**Published:** 2018-03-22

**Authors:** Enrique Casalino, Aiham Ghazali, Donia Bouzid, Stephanie Antoniol, Laurent Pereira, Philippe Kenway, Christophe Choquet, and the Emergency Department study group on respiratory viruses

**Affiliations:** 1 Emergency Department, Hôpital Bichat, Assistance Publique-Hôpitaux de Paris (AP-HP), Paris, France; 2 Université Paris Diderot, Sorbonne Paris Cité, EA 7334 « Recherche clinique coordonnée ville-hôpital, Méthodologies et Société (REMES) », Paris, France; 3 Study Group for Efficiency and Quality of Emergency Departments and Non-Scheduled Activities Departments, Paris, France; University of Washington, UNITED STATES

## Abstract

**Objectives:**

Influenza vaccination (IV) coverage remains low in France. Objectives were to assess patient knowledge and behaviors and missed opportunities for vaccination (MO) and their impact on vaccine uptake.

**Methods:**

This is a prospective-observational study, including emergency department patients at risk for severe influenza. Patients were interviewed about their knowledge and behaviors. We evaluated the health-care voucher scheme (HCVS) and MO.

**Results:**

868 patients were included. Vaccine uptake was 33.2%, 42% of patients knew about the possible severity of influenza, 23% thought that they were not at risk for severe influenza, 39% knew that they have an indication for the vaccine, and 4.3% to 11.5% expressed reservations concerning IV side effects and effectiveness. HCVS was used by 44.3% of patients, but only 14.8% had been vaccinated. MO were reported by 484 patients (69.4%) declaring 1104 consultations and 148 IV propositions (86.6%). Predictors of vaccine uptake (p<0.0001) were: knowledge of serious and fatal influenza forms [OR 0.36 (CI95% 0.25–0.5)]; confidence in influenza vaccine effectiveness [0.38 (0.2–0.7)]; opposition to vaccines [0.22 (0.1–0.48)]; visit to general practitioner [4.53 (2.9–7.1)]; general practitioner proposed IV [2.1 (1.2–3.4)].

**Conclusion:**

Our results indicate that high rate of missed opportunities, some patient behaviors and general practitioner visits may explain low influenza vaccine uptake, and that HCVS use is a complex process. Of interest, we found that the patient’s knowledge of the potential severity of influenza is not sufficient to promote vaccine, suggesting that the information strategy must be adapted to each patient behavior.

## Introduction

The seasonal epidemic influenza remains a major public health issue with 3–5 million severe cases, resulting in up to 650 000 deaths annually [1]. Every winter the seasonal flu affects 2 to 8 million people in France, causing several thousand deaths mainly elderly people or patients with chronic diseases, both of which are currently accepted indications for influenza vaccination [2]. Current recommendations insist on the importance of vaccination, which is currently considered as the most effective way to prevent the disease [1–4]. Worldwide influenza vaccination coverage ranges from 10% to 80% for the populations at risk of severe forms [5–7]. The reasons behind these variations are insufficiently identified [7,8]. Studies have indicated that among the possible explanations are certain healthcare attitudes and behaviors, health information provided by health care workers, especially general practitioners, patients’ underlying clinical conditions, individual perceptions regarding the harms and benefits of vaccination and, even, household contacts attitudes [9–13]. Patient’s hesitancy is probably an important factor [14] but it is highly different from one country or region to another. Furthermore, it has been reported that missed opportunities to vaccinate are frequently associated with low vaccination rates [15,16].

To our knowledge, the factors associated with influenza vaccination coverage in the population at risk of severe flu have not been described in France, apart from some studies in health care workers [17,18]. We hypothesized that patient’s behaviors and missed opportunities (MO) may be associated with influenza vaccine uptake. To our knowledge there presently exists no data evaluating influenza vaccination coverage and patient behaviors and missed opportunities in an emergency department setting.

## Objectives

The objectives of this study were to estimate the influenza vaccine uptake during the 2016–2017 seasonal epidemic period of high-risk patients for severe influenza among emergency department admissions, to describe patient behaviors and missed opportunities distribution among them, and to identify predictors of seasonal influenza vaccine uptake.

## Methods

### Type and study period

In France, the influenza vaccination period began on October 6, 2016, when the vaccine was available, and the influenza vaccination was authorized until January 31, 2017. This study began on December 5, 2017 and lasted 8 weeks until January 31, 2017, deadline for influenza vaccination in France [19]. The French National Institute of Health Surveillance announced the start of the influenza outbreak period on December 12, 2016. Therefore, our study started when we have considered that the influenza vaccine uptake had reached its plateau or that subsequent increase would have been very low [6].

### Setting

Bichat hospital is in Paris area. It serves northern districts of Paris and its nearest suburb, with a population of more than 350,000 inhabitants. The population is characterized by a low income and poor primary care availability [20], a higher rate of immigrant population and unemployment than the region averages [21,22]. Bichat emergency department treats more than 80,000 patients per year.

### Population of the study

Participation in the study was proposed to all the adult patients aged over 18 years and with an underlying clinical condition that indicates the influenza vaccine according to WHO and French authorities recommendations [4,19], identified by the emergency team: i) individuals aged equal or more than 65 years; ii) individuals with chronic medical conditions; iii) pregnant women at any stage of pregnancy. Patients with life-threatening conditions, cognitive disorder and non-French speaking were excluded from the study.

### Interventions and definitions

A questionnaire was administered to the patients who accepted to participate in our study. The questionnaires were complemented by direct questioning of the patients by health care workers of the emergency department. This questionnaire evaluated the presence of underlying clinical conditions indicating the influenza vaccine [23]: age ≥65 years, asthma, bronchopulmonary dysplasia, cystic fibrosis, chronic respiratory failure, cardiac failure, cardiac valvulopathy, congenital heart disease, cardiovascular disease, renal failure, nephrotic syndrome, sickle-cell anemia, hepatic failure, diabetes, systemic corticosteroid therapy, leukemia or lymphoma, immunosuppression, cancer, HIV infection, obesity and pregnancy; history of the influenza vaccine for the current year and for the previous epidemic periods; to evaluate missed opportunities, we asked patients about the number of outpatient consultations with their general practitioner or specialist physicians and number of hospital stays, and the number of influenza vaccine proposals during these consultations or hospitalizations, during the vaccination period from October 6, 2016 to January 31, 2017; patient attitudes, concerns and behaviors about influenza vaccination.

In France, Social Security (health insurance system) systematically sends, as soon as the influenza vaccine is available, a letter to all insured persons with a clinical condition that is at increased risk of developing a severe form of influenza. This letter is sent every year to 12 million people [24]. The letter reminds the patient that the vaccine is recommended and allows the patient to receive the vaccine free of charge. The letter must be signed by a physician and the patient must then retrieve the vaccine from the chemist. The vaccination can be given by the general practitioner or by a nurse. To evaluate what role the voucher system plays in uptake of influenza vaccine, patients were questioned about the reception of this letter and every stage of the process until the vaccination.

Influenza vaccine uptake during the current 2016–2017 influenza epidemic period was defined as the rate of patients vaccinated between October 6, 2016 and the date of emergency department attendance. In France, influenza vaccine indications are all the clinical conditions at risk of developing severe forms of influenza [25].

### Statistical analysis

Continuous variables are presented using mean ± standard deviation (SD) and categorical variables such as absolute number and percentage of the total (%). To assess the association between vaccinated and non-vaccinated groups we used multiple logistic regression. Variables that p<0.2 were included in the multivariate stepwise logistic regression model to determine those independently related to influenza vaccination coverage. Statistica 12® (StatSoft) software was used for data collection and analysis.

### Ethics statement

The dataset is currently used as an ED quality measure of influenza vaccination prescription in the context of on-going emergency activity and performance evaluation. The protocol was conducted in agreement with the Helsinki declaration. This dataset was completely anonymous and did not contain any identifiable personal health information. The protocol was approved by the Emergency Department committee on ethics, research and informatics. In accordance with the instructions of the ethics committee, the patients were informed by flyer and by posters in the service. Emergency staff gave them an explanatory document. Then, agreement of the patients was obtained verbally. This information was recorded in the patient’s records.

## Results

**Fig 1:**
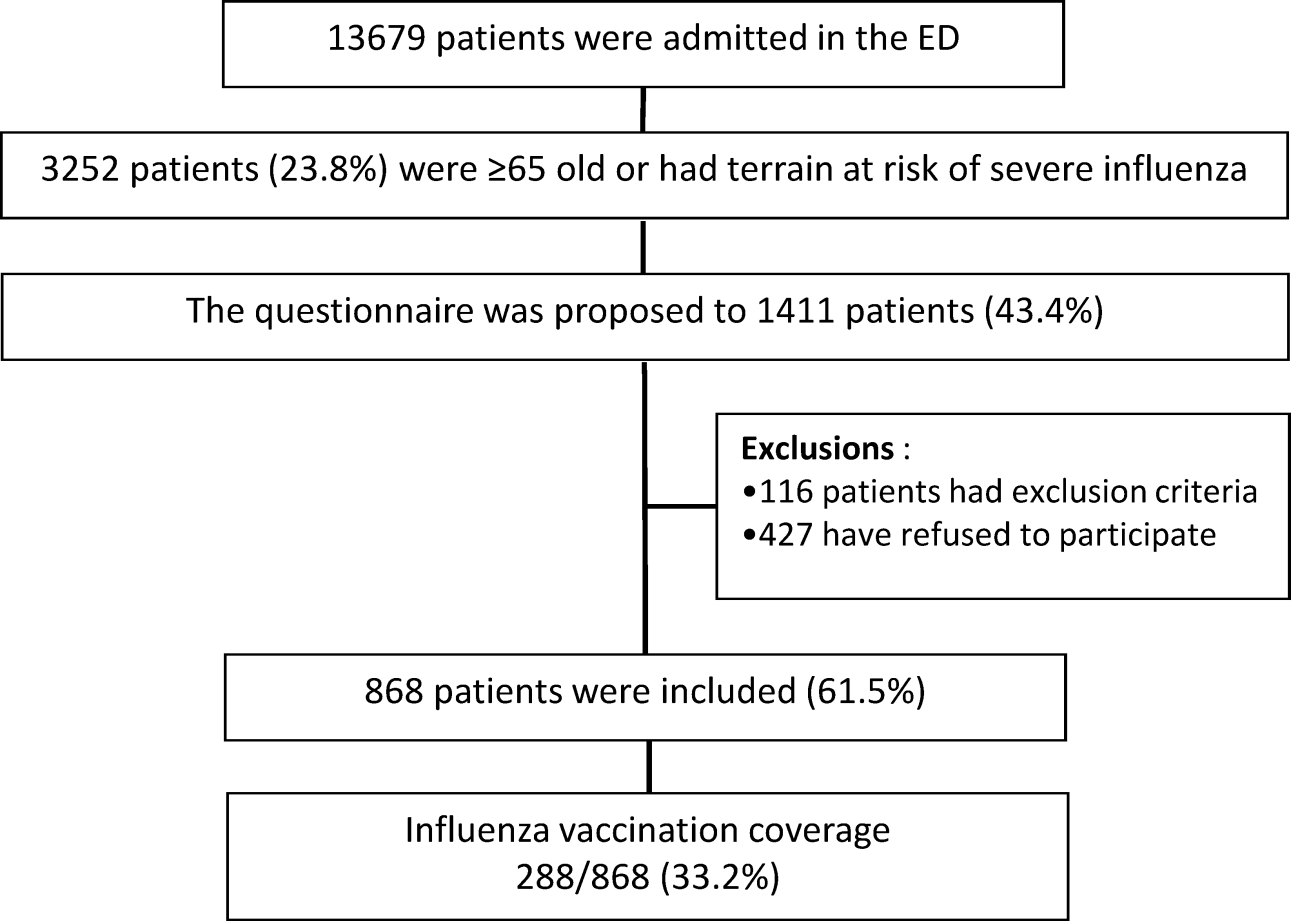
Study flowchart.

During the study period, 13,679 patients were admitted in the ED. We estimated based on the encoding and the categories of age, that 3,252 patients (23.8%) were ≥65 years old or had an underlying clinical condition at risk of severe influenza and that indicated influenza vaccine. The questionnaire was proposed to 1,411/3,252 patients (43.4%): 116 patients had exclusion criteria and 427 refused to participate. All in all, 868 patients accepted to participate and completed the questionnaire (Fig 1: Study flowchart).

### Description of the study population

The main characteristics of the patients and their influenza vaccination history are presented in Table 1. Patient behaviors, concerns and attitudes concerning the vaccination are presented in Table 2.

**Table 1:** Main characteristics of the study population.

	n	%
	(mean±DS)	
Age	58±36	
Sex		
Male	390	44.9
Female	478	55.1
**Underlying clinical conditions**		
Age ≥ 65 years	666	76.4
Diabetes	237	27.2
Human Immunodeficiency virus infection	13	1.5
Previous history of respiratory illness	193	22.1
Asthma	120	14
COPD	43	5
Other	18	2.1
Previous cardiovascular history	250	26.7
Coronary heart disease	77	8.9
Cardiac failure (or cardiac insufficiency)	45	5.2
Serious heart rhythm disturbances	42	4.8
Other	72	8.3
Previous history of underlying neurological disorders	41	4.7
Stroke	18	2.1
Myopathy, myasthenia, Charcot disease	5	0.6
Kidney failure, kidney diseases	28	3.2
Obesity	35	4
Pregnancy	0	0
**History of influenza vaccination**		
Vaccinated every year	239	27.4
Vaccinated during this seasonal epidemic flu	288	33
Vaccinated during the past seasonal epidemic flu	343	39.2
Vaccinated at least one time (or once)	423	48.5

**Table 2:** Beliefs about influenza vaccine.

	n	%
**Representations concerning the vaccination**		
Are you worried about the side effects of the influenza vaccination?	118	13.6
Have you already had some side effects after an influenza vaccination?	66	7.6
Do you think that the influenza vaccine is contraindicated in your case?	32	4.3
Do you think that the influenza vaccine is ineffective?	118	13.6
Are you generally reluctant or opposed to vaccines?	96	11
**Knowledge of the indication of the vaccine**		
Do you know that influenza can be severe and in some cases deadly?	368	42.2
I consider that I am not at risk of developing a severe form of flu	206	23.6
Do you know that the influenza vaccine is indicated in your case?	344	39.4

### Evaluation of the voucher sent by the health insurance system

In total, 386/868 (44.5%) patients declared that they had received a voucher from Social Security; 123/386 (31.9%) reported validating the voucher by their doctor; 115/123 (93.5%) reported having collected the vaccine at the chemist; 57/115 (49.6%) reported having been vaccinated. Finally, of the 288 patients that declared having been vaccinated during the current epidemic, 57 were vaccinated via this process (57/288 (19.8%)). Results are presented in Table 3.

**Table 3:** Evaluation of the voucher of health insurance system and missed opportunities.

	n	%
**Voucher from the social health insurance system**		
Declared they had received the letter from the social security	386	44.5
Declared they had asked their GP to sign the letter and stamp it	123	31.9
Declared they had received the vaccine at the chemist	115	29.8
Declared they had been vaccinated	57	14.8
**Consultations and hospitalizations declared by the patients**		
Consultation with their general practitioner	307	35.2
Declared number of consultations	606	1.9±0.9
The influenza vaccination was recommended?	111	36.2
Consultation with a specialist	192	22
Declared number of consultations	398	2±1.7
The influenza vaccination was recommended?	19	9.9
Out-patient hospital consultation	100	11.5
The influenza vaccination was recommended?	18	18
Hospitalization	54	6.2
The influenza vaccination was recommended?	4	7.4

### Evaluation of missed opportunities

In all, 1108 consultations were reported, and 484 patients (55.8%) reported having had at least one consultation. The vaccine was recommended by the doctor in 148 cases. Rates of missed opportunities were 69.4% (336/484) and 86.6% (956/1104). Although most patients had more than one consultation during this period, those who received an offer received it on only one occasion rather than at each visit. Table 3 presents the details of the answers depending on the type of consultations and hospitalizations.

### Determinants of influenza vaccine uptake

Influenza vaccine uptake was 288/868 (33.2%). As presented in Table 4, several variables were associated in univariate analysis with influenza vaccine uptake. On multivariate analysis, significant predictors of influenza vaccine uptake were: knowledge that the flu can be serious and even fatal [OR 0.36 (CI95% 0.25–0.5), p = 0.000001]; confidence in influenza vaccine effectiveness [OR 0.38 (0.2–0.7), p = 0.001]; opposition to vaccines in general [OR 0.22 (0.1–0.48), p = 0.0001]; the patient visited his general practitioner [OR 4.53 (2.9–7.1), p = 0.000001]; the general practitioner recommended the vaccine [OR 2.1 (1.2–3.4), p = 0.006].

**Table 4:** Predictors of influenza vaccine uptake.

	unvaccinated	vaccinated		Multiple Logistic Regression		
	n (%)	n (%)	P	OR	IC95%	P
**Underlying conditions for severe influenza**						
Age ≥65 years	423 (63.5%)	243(36.5%)	0.00009			
Diabetes	145 (61.2%)	92 (38.8%)	0.02			
Human immunodeficiency virus infection	9 (55.6%)	4 (44.4%)	0.7			
Respiratory diseases	143(74.1%)	50 (25.9%)	0.02			
Cardiovascular diseases	171 (68.4%)	79 (31.6%)	0.6			
Neurologic diseases	29 (70.7%)	12 (29.3%)	0.6			
Renal diseases	24 (85.7%)	4 (14.3%)	0.03			
Obesity	17 (48.6%)	18 (51.4%)	0.02			
**History of influenza vaccination**						
Vaccination earlier epidemic period	208 (60.6%)	135 (39.4%)	0.001			
Vaccinated at least once	298 (70.5%)	125 (29.6%)	0.03			
Annual vaccination	132 (55.2%)	107 (44.8%)	0.00001			
**Patients knowledge and behaviors**						
Fears about the side effects of the vaccine	51 (77.3%)	15 (22.7%)	0.06			
Knowledge of having an indication for the vaccine	253 (73.6%)	91 (26.5%)	0.0009			
Knowledge that the flu can be serious and even fatal	280 (76.1%)	88 (23.9%)	0.000001	0.36	0.25–0.5	0.000001
Do you think that you are not at risk of severe flu	141 (68.5%)	65 (31.6%)	0.6			
Confidence in influenza vaccine effectiveness	99 (83.9%)	19 (16.1%)	0.00003	0.38	0.2–0.7	0.001
Fears about the side effects of the vaccine	32 (100%)	0 (0%)	0.00005			
Opposition to vaccines in general	88 (91.7%)	8 (8.3%)	0.000001	0.22	0.1–0.48	0.0001
**Voucher of Health insurance system**						
Received the voucher of Health Insurance System	258 (66.8%)	128 (33.2%)	0.9			
The patient visited his General Practitioner	49 (39.8%)	74 (60.2%)	0.000001	4.53	2.9–7.1	0.000001
The patient recovered the vaccine in pharmacy	40 (34.8%)	75 (65.2%)	0.000001			
**Missed opportunities**						
General practitioner seen in the past 3 months	223 (72.6%)	84 (27.4%)	0.009			
General practitioner recommended the vaccine	65 (58.6%)	46 (41.4%)	0.04	2.1	1.2–3.4	0.006
Consultation with his specialist in the past 3 months	255 (88.5%)	33 (11.5)	0.000001			
Specialist recommended the vaccine	10 (52.6%)	9 (47.4%)	0.2			
Hospital consultation in the past 3 months	82 (82%)	18 (18%)	0.0007			
Physician recommended the vaccine	14 (77.8%)	4 (22.2%)	0.3			
Hospitalization in the past 3 months	54 (100%)	0 (0%)	0.000001			
Physician recommended the vaccine						

## Discussion

Our study shows that up to 24% of the patients admitted in the emergency department during the early- and epidemic seasonal influenza period were at high risk for severe influenza, and that influenza vaccine uptake was only 33% among them. We found that the concerns expressed by the patients about vaccine side effects and inefficacy were infrequent (4.3% to 11%), and that 42% of the patients knew that influenza can be serious and even deadly. Our results indicate that 39.4% of the patients declared that they knew they had an indication for the vaccination; however 23.6% of them thought they were not at risk for a severe form of influenza. Our study highlights the fact that missed opportunities for influenza vaccination were very high, 69.4% of patients and 86.6% of consultations. Our results also indicate that influenza vaccine uptake was strongly associated with patients concerns and behaviors and the proposal of influenza vaccination by the general practitioner.

In the present study only 27.2% of the included patients declared that they had been vaccinated every year, 39.2% that they were vaccinated during the last epidemic season, and 48.2% declared that they had been vaccinated at least once in their lives. The rate of influenza vaccine uptake among patients visiting the emergency department during the 2016 influenza season was 33.2%. These figures are lower than the national rate of influenza vaccination coverage in France, which is 49% [26] that is very close to those reported in some western countries [5–7,27,28]. The characteristics of the catchment area population of the hospital, which are poor urban areas and low primary care availability, may explain these results [29].

### Patient’s behaviors and knowledge

We have analyzed behaviors, concerns and attitudes of the patients regarding the influenza vaccination. And we have observed that less than 15% of patients expressed reservations about influenza vaccine effectiveness and concerns about its side effects. Similarly, only 11% of patients expressed worries on the usage of vaccination in general, while unfavorable attitudes toward pandemic influenza vaccination have been reported in France in up to 50% of patients [30]. Our results are quite encouraging about the seasonal influenza vaccination. Nevertheless, up to 60% of our patients underestimated the risk of severe influenza, 60% were unaware of having an indication for the influenza vaccination, and 24% underestimated their own risk for presenting a severe or deadly form of seasonal influenza. These data suggest an increased need for information on influenza vaccine indications.

### Voucher effectiveness

Among the missed opportunities, we have evaluated the French health insurance system process. Only 44.3% of the patients declared that they had received an Influenza vaccine voucher. Of course, under-reporting by patients is possible. But it could also be the case that not all patients aged between 15 and 64 years with a chronic underlying clinical condition were declared to the social security by their practitioner as being at risk. In the normal procedure, the patient must ask his general practitioner to sign and stamp the voucher, which means that the patient is the main actor in the activation of the procedure. Once the patient has retrieved the signed and stamped voucher, he can retrieve the vaccine in a dispensing chemist and, finally, be vaccinated by a health care worker. This clinical pathway is complex and our study shows that only 14.8% of the patients who had received the letter went through the entire procedure and were vaccinated. Among all the patients that had been vaccinated, only 19.8% of them had followed this procedure. Our study shows two critical points in this procedure. Firstly, the patient must take an appointment with the general practitioner to activate the procedure, which only 31.9% declared having done after receiving the voucher. Since two years, the process has been modified and the chemist can deliver the vaccine in the already vaccinated patients. Secondly, when we look at the actual rate of the vaccine uptake, we found that only 49.9% of the patients having retrieved the vaccine had been vaccinated. Vaccination by chemist is being tested in two regions of France. Our results confirm the need for a simplification of the vaccination process in these patients at high risk of severe forms of influenza. Our results indicate that most of the vaccinated patients (80%) were vaccinated mainly by their doctor or were able to access the vaccine because they had already been vaccinated. In both cases, vaccine is free.

### Missed opportunities

Our results indicate that the number and percentage of missed opportunities are very high (66.9% of patients and 86.6% of consultations), and close like those recently reported in outpatients context [27]. They show that even if he sees his general practitioner or a different specialist in or out of the hospital, the patient only receives one proposition for the vaccination. It is interesting to notice that the rate of proposals for the vaccination by the general practitioner was higher than that by the specialist, and that the proposal rate during a hospital stay was the lowest of all. Our results indicate the need to modify the procedures of influenza vaccination and identify the barriers to vaccination during ambulatory care and notably during hospital stay.

Our study reveals that among the studied variables, many of them were associated in univariate analysis with influenza vaccine uptake. For instance, patients over the age of 65 were more frequently vaccinated than younger patients with chronic health conditions, which corresponds to previous studies [15,13,27]. We have also found out that patients who were previously vaccinated were more often vaccinated during the current epidemic period. It has been recently reported that regular vaccination was associated with higher seasonal influenza vaccine uptake in people at risk [31]. Regarding the significant predictors of influenza vaccine uptake in multivariate analysis, our results are consistent with previous publications concerning the doubts on the usefulness and safety of the vaccine [28,32], and they highlight the impact on influenza vaccine uptake of patients' fears on the side effects and confidence on vaccine effectiveness, and their opposition to vaccines. We found, in opposition with previous studies among outpatients, health care personal and general population [28–32], that the knowledge of the risk of severe and even deadly forms of influenza was associated with a reduced rate of vaccination. This appears somewhat surprising, insofar as knowledge of the risks of the severe form and the perceived importance of vaccination are at the basis of most campaigns of vaccination [33]. However, and rather paradoxically, some campaigns may also have a negative impact on population’s health attitudes and behaviors that could be linked to misleading or wrong information [34]. We have not explored this type of barriers, but it is likely that many different psychological, contextual, socio-demographic, personality characteristics such as risk aversion and physical barriers can help to explain these results [32,35,36]. We believe that information strategies and vaccination campaigns need to be adapted to the characteristics of the targeted population. The population of our study has particular characteristics that can explain this result and the need for specific information tools.

### Study strengths and limitations

The study was conducted in an emergency department that serves an urban area with low primary care availability and characterized by an unfavorable social and economic context. Thus, our population does not reflect the whole population. Data collection was done from a questionnaire done by an emergency department doctor or nurse. It was not obtained from medical records or electronic data. We might have over or underestimated influenza vaccine uptake. Nevertheless, there was a sufficient inclusion rate and the number of patients included in this study was important which gives validity to our calculations and results.

## Conclusions

Our study indicates that influenza vaccine uptake in the northern districts of Paris remains low. We have looked at the patient behaviors and concerns associated with the influenza vaccine uptake, and we have found that the knowledge of the risk of severe forms of the influenza is not associated with a better influenza vaccine uptake. New studies will be needed to better understand patient-specific dimensions and patient management of risk knowledge. Our study indicates that missed opportunities are very frequent and that the role of the patient and general practitioner remain essential. We have also identified key points in current vaccination strategy in France, specifically the poor coverage of people at risk and the complexity of the procedure leading to vaccination. We believe that the implementation of vaccination campaigns should be based on strategies tailored to the fears and behaviors of each patient.

## References

[1] Iuliano AD, Roguski KM, Chang HH, Muscatello DJ, Palekar R, Tempia S, et al Estimates of global seasonal influenza-associated respiratory mortality: a modelling study. Lancet. 2017; pii: S0140-6736(17)33293-2. 10.1016/S0140-6736(17)33293-2 PMC5935243 29248255

[2] Haut Conseil de la santé publique. Avis relatif à l’efficacité de la vaccination contre la grippe saisonnière notamment chez les personnes âgées et à la place de la vaccination des professionnels de santé dans la stratégie de prévention de la grippe. 2014. www.hcsp.fr/explore.cgi/avisrapportsdomaine?clefr=424 (accessed October 10, 2017)

[3] Bonmarin I. Influenza activity in mainland France, season 2015–2016. BEH. 2016;32–33:558–63. http://www.invs.sante.fr/beh/2015/32-33/2015_32-33_4.html (accessed July 10, 2017)

[4] Grohskopf LA, Sokolow LZ, Broder KR, Olsen SJ, Karron RA, Jernigan DB, et al Prevention and Control of Seasonal Influenza with Vaccines. MMWR Recomm Rep. 2016;65(5):1–54. doi: 10.15585/mmwr.rr6505a1 27560619 10.15585/mmwr.rr6505a1

[5] European Centre for Disease Prevention and Control. Influenza. 2016. Available from: http://ecdc.europa.eu/en/healthtopics/influenza/Pages/home.aspx (accessed June 3, 2017).

[6] Centers for Disease Control and Prevention. Flu vaccination coverage. United States, 2014–15 influenza season. http://www.cdc.gov/flu/fluvaxview/coverage-1415estimates.htm (accessed July 12, 2017).

[7] Palache A, Oriol-Mathieu V, Abelin A, Music T, Influenza Vaccine Supply task force (IFPMA IVS). Seasonal influenza vaccine dose distribution in 157 countries (2004–2011). Vaccine. 2014;32(48):6369–76. doi: 10.1016/j.vaccine.2014.07.012 25442403 10.1016/j.vaccine.2014.07.012

[8] Doherty M, Schmidt-Ott R, Santos JI, Stanberry LR, Hofstetter AM, Rosenthal SL, et al Vaccination of special populations: Protecting the vulnerable. Vaccine. 2016;34(52):6681–6690. doi: 10.1016/j.vaccine.2016.11.015 27876197 10.1016/j.vaccine.2016.11.015

[9] Wagner AL, Montgomery JP, Xu W, Boulton ML. Influenza vaccination of adults with and without high-risk health conditions in China. J Public Health (Oxf). 2017;39(2):358–365. 27160858 10.1093/pubmed/fdw041

[10] Blank PR, Bonnelye G, Ducastel A, Szucs TD. Attitudes of the general public and general practitioners in five countries towards pandemic and seasonal influenza vaccines during season 2009/2010. PLoS One. 2012;7(10):e45450 doi: 10.1371/journal.pone.0045450 23071519 10.1371/journal.pone.0045450 PMC3469560

[11] Bödeker B, Remschmidt C, Schmich P, Wichmann O. Why are older adults and individuals with underlying chronic diseases in Germany not vaccinated against flu? A population-based study. BMC Public Health. 2015;15:618 doi: 10.1186/s12889-015-1970-4 26148480 10.1186/s12889-015-1970-4 PMC4492002

[12] Privileggio L, Falchi A, Grisoni ML, Souty C, Turbelin C, Fonteneau L, et al Rates of immunization against pandemic and seasonal influenza in persons at high risk of severe influenza illness: a cross-sectional study among patients of the French Sentinelles general practitioners. BMC Public Health. 2013;13:246 doi: 10.1186/1471-2458-13-246 23514534 10.1186/1471-2458-13-246 PMC3621692

[13] Taylor E, Atkins KE, Medlock J, Li M, Chapman GB, Galvani AP. Cross-Cultural Household Influence on Vaccination Decisions. Med Decis Making. 2016;36(7):844–53. doi: 10.1177/0272989X15591007 26085600 10.1177/0272989X15591007 PMC4683113

[14] Schmid P, Rauber D, Betsch C, Lidolt G, Denker ML. Barriers of Influenza Vaccination Intention and Behavior—A Systematic Review of Influenza Vaccine Hesitancy, 2005–2016. PLoS One. 2017 26;12(1):e0170550 doi: 10.1371/journal.pone.0170550 28125629 10.1371/journal.pone.0170550 PMC5268454

[15] Djibo DA, Peddecord KM, Wang W, Ralston K, Sawyer MH. Factors Associated With Missed Opportunities for Influenza Vaccination: Review of Medical Records in a Diverse Sample of Primary Care Clinics, San Diego County, 2010–2011. J Prim Care Community Health. 2015;6(3):147–53. doi: 10.1177/2150131914559541 25432588 10.1177/2150131914559541

[16] Williams WW, Lu PJ, O'Halloran A, Kim DK, Grohskopf LA, Pilishvili T, et al Surveillance of Vaccination Coverage among Adult Populations—United States, 2015. MMWR Surveill Summ. 2017;66(11):1–28. doi: 10.15585/mmwr.ss6611a1 28472027 10.15585/mmwr.ss6611a1 PMC5829683

[17] Hulo S, Nuvoli A, Sobaszek A, Salembier-Trichard A. Knowledge and attitudes towards influenza vaccination of health care workers in emergency services. Vaccine. 2017 1 5;35(2):205–207. doi: 10.1016/j.vaccine.2016.11.086 27919630 10.1016/j.vaccine.2016.11.086

[18] Bouadma L, Barbier F, Biard L, Esposito-Farèse M, Le Corre B, Macrez A, et al Personal decision-making criteria related to seasonal and pandemic A(H1N1) influenza-vaccination acceptance among French healthcare workers. PLoS One. 2012;7(7):e38646 doi: 10.1371/journal.pone.0038646 22848342 10.1371/journal.pone.0038646 PMC3407215

[19] Assurance Maladie. Grippe saisonnière. La campagne nationale de vaccination contre la grippe saisonnière se déroule du 6 octobre 2016 au 31 janvier 2017. Le point sur la campagne, le dispositif mis en place et les modalités pratiques. 2016. http://www.ameli.fr/professionnels-de-sante/infirmiers/exercer-au-quotidien/vaccination-contre-la-grippe-saisonniere/la-campagne-de-vaccination-2016-2017.php (accessed October9, 2017)

[20] Agence Régionale de santé. Etat de santé et inégalités sociales et territoriales: éléments de diagnostic francilien. 2011. http://www.ars.iledefrance.sante.fr/fileadmin/ILE-DE-FRANCE/ARS/1_Votre_ARS/3_Nos_Actions/3_PRS/Note_diagnostic_ISTS_V9_180111.pdf (accessed September 19, 2017)

[21] Brutel C. La localisation géographique des immigrés. Une forte concentration dans l’aire urbaine de Paris. Insee Statistiques. 2016;1591 https://www.insee.fr/fr/statistiques/2121524 (accessed September 19, 2017)

[22] Caenen Y, Decondé C, Jabot D, Martinez C, Ouardi S, Eloy P, Jouny L. Une mosaïque sociale propre à Paris. 2017;53 https://www.insee.fr/fr/statistiques/2572750 (accessed October 9, 2017).

[23] Shah NS, Greenberg JA, McNulty MC, Gregg KS, Riddell J, Mangino JE, et al Severe Influenza in 33 US Hospitals, 2013–2014: Complications and Risk Factors for Death in 507 Patients. Infect Control Hosp Epidemiol. 2015;36(1):1251–60. 26224364 10.1017/ice.2015.170

[24] Santé Publique France. Lancement de la campagne de vaccination contre la grippe saisonnière. 2017; http://www.veille-infosplus.fr/filagenda/files/2017/10/DP-Grippe-2017-VDEF.pdf (accessed October 9, 2017)

[25] Direction générale de la santé. Comité technique des vaccinations. Vaccination contre la grippe saisonnière. Guide des vaccinations Édition 2012. http://inpes.santepubliquefrance.fr/10000/themes/vaccination/guide-vaccination-2012/pdf/GuideVaccinations2012_Vaccination_contre_la_grippe_saisonniere.pdf

[26] Nicand E. Couverture vaccinale en France en 2014: un bilan contrasté. 2015 Available from: https://www.mesvaccins.net/web/news/6140-couverture-vaccinale-en-france-en-2014-un-bilan-contraste (accessed October 1st, 2017)

[27] Lu PJ, O'Halloran A, Ding H, Srivastav A, Williams WW. Uptake of Influenza Vaccination and Missed Opportunities Among Adults with High-Risk Conditions, United States, 2013. Am J Med. 2016; 129(6):636.e1–636.e11. 10.1016/j.amjmed.2015.10.031 PMC5831078 26551981

[28] Vila-Candel R, Navarro-Illana P, Navarro-Illana E, Castro-Sánchez E, Duke K, Soriano-Vidal FJ, et al Determinants of seasonal influenza vaccination in pregnant women in Valencia, Spain. BMC Public Health. 2016;16(1):1173 doi: 10.1186/s12889-016-3823-1 27871262 10.1186/s12889-016-3823-1 PMC5117491

[29] Cheng PY, Palekar R, Azziz-Baumgartner E, Iuliano D, Alencar AP, Bresee J, et al Burden of influenza-associated deaths in the Americas, 2002–2008. Influenza Other Respir Viruses. 2015;9(Suppl 1):13–21. 26256291 10.1111/irv.12317 PMC4549098

[30] Peretti-Watel P, Verger P, Raude J, Constant A, Gautier A, Jestin C, et al Dramatic change in public attitudes towards vaccination during the 2009 influenza A(H1N1) pandemic in France. Euro Surveill. 2013;18(44). pii: 20623. 10.2807/1560-7917.es2013.18.44.20623 24176658

[31] Adadan Güvenç I, Parıldar H, Şahin MK, Erbek SS. Better knowledge and regular vaccination practices correlate well with higher seasonal influenza vaccine uptake in people at risk: Promising survey results from a university outpatient clinic. Am J Infect Control. 2017;pii:S0196-6553(17)30197-9. 10.1016/j.ajic.2017.02.041 28449918

[32] Schmid P, Rauber D, Betsch C, Lidolt G, Denker ML. Barriers of Influenza Vaccination Intention and Behavior—A Systematic Review of Influenza Vaccine Hesitancy, 2005–2016. PLoS One. 2017;12(1):e0170550 doi: 10.1371/journal.pone.0170550 28125629 10.1371/journal.pone.0170550 PMC5268454

[33] Meyer SB, Lum R. Explanations for Not Receiving the Seasonal Influenza Vaccine: An Ontario Canada Based Survey. J Health Commun. 2017;22(6):506–514. doi: 10.1080/10810730.2017.1312720 28448208 10.1080/10810730.2017.1312720

[34] Rosselli R, Martini M; Fluad Effect Working Group, Bragazzi NL, Watad A. The Public Health Impact of the So-Called "Fluad Effect" on the 2014/2015 Influenza Vaccination Campaign in Italy: Ethical Implications for Health-Care Workers and Health Communication Practitioners. Adv Exp Med Biol. 2017; 973:125–134. doi: 10.1007/5584_2017_39 28452003 10.1007/5584_2017_39

[35] Massin S, Ventelou B, Nebout A, Verger P, Pulcini C. Cross-sectional survey: risk-averse French general practitioners are more favorable toward influenza vaccination. Vaccine. 2015;33(5):610–4. doi: 10.1016/j.vaccine.2014.12.038 25545596 10.1016/j.vaccine.2014.12.038

[36] Ward J, Raude J. Understanding influenza vaccination behaviors: a comprehensive sociocultural framework. Expert Rev Vaccines. 2014;13(1):17–29. doi: 10.1586/14760584.2014.863156 24308575 10.1586/14760584.2014.863156

